# Exploring Gaps in Maternal WASH Practices: A Cross‐Sectional KAP Study in Rural Coastal Bangladesh

**DOI:** 10.1002/puh2.70171

**Published:** 2025-11-29

**Authors:** Prantu Sen, Mohammad Asadul Habib, Tanjina Rahman, Akibul Islam Chowdhury, Kazi Muhammad Rezaul Karim

**Affiliations:** ^1^ Department of Food Technology and Nutrition Science Noakhali Science and Technology University Noakhali Bangladesh; ^2^ Institute of Nutrition and Food Science University of Dhaka Dhaka Bangladesh; ^3^ Department of Nutrition and Food Engineering Daffodil International University Savar Bangladesh

**Keywords:** attitude, knowledge, practice, sanitation, water accessibility

## Abstract

**Aims:**

This study explored the knowledge, attitudes, and practices (KAPs) related to water, sanitation, and hygiene (WASH) and food safety among mothers in Noakhali, a coastal district of Bangladesh.

**Methods:**

A cross‐sectional study involving 325 mothers was conducted in the Noakhali district of Bangladesh using purposive sampling. A standard pretested questionnaire was used to assess KAPs regarding WASH practices.

**Results:**

Research findings indicated that although most participants had optimal knowledge, only a smaller percentage displayed positive attitudes and proper hygiene practices. Mothers with higher education and better economic standing were significantly more likely to exhibit favorable WASH behaviors, underscoring the critical role of education and economic empowerment. Importantly, mothers with good knowledge were nearly 10 times (adjusted odds ratio [AOR]: 9.621, 95% confidence interval [CI]: 4.23–21.88) more likely to practice safe hygiene, revealing the transformative potential of informed communities.

**Conclusion:**

This study calls for culturally appropriate, community‐driven interventions that honor the strength and resilience of rural Bangladeshi women. Promoting education, improving infrastructure, and expanding awareness through schools, media, and grassroots initiatives can pave the way for healthier, more dignified lives. Empowering mothers with knowledge and tools such as educational resources, training programs, and healthcare services is not only a public health necessity but also an investment in the future of rural Bangladesh.

## 1 Introduction

Access to safe water, sanitation, and hygiene (WASH) is essential for human health and dignity, and it is a fundamental human right. Despite global progress, approximately 1.8 billion people worldwide still drink water contaminated with feces [1], and over 2.3 billion lack even the most basic sanitation services [2]. However, according to the World Health Organization (WHO) [3], each year, unhygienic food causes 600 million illnesses and 420,000 deaths worldwide [3]. According to previous studies, inadequate level of knowledge, attitude, and practice (KAP) towards the consequences of unsafe water, poor sanitation and hygiene practice, a lack of basic sanitary facilities, and carelessness with food handling are the main contributors to poor growth and development of a nation [4, 5].

The WHO and international development frameworks underscore the interplay between environmental conditions and behavioral practices in tackling the WASH crisis [6]. In many developing countries, such as rural and coastal areas, inadequate infrastructure, limited resources, and socio‐behavioral discrepancies contribute to the ongoing vicious cycle of disease transmission and undernutrition. Diarrheal disease, one of the direct consequences of unsafe WASH, is the second leading cause of death among children under five globally [7]. An alarming number of these deaths occur in South and Southeast Asia, where childhood morbidity and mortality are still high [8].

In a developing country like Bangladesh, ensuring WASH across all regions is a challenge and contributes to poor health and development. Coastal areas of Bangladesh are one of the primary victims in this case, mainly because of the lack of drinkable water, insufficient sanitation, and poor hygiene behavior and practice [9]. Low levels of awareness, poverty, and limited maternal education further worsen WASH practices in these communities. As primary caregivers, mothers play a key role in shaping household hygiene behaviors. They are burdened with cooking, child care, sanitation management, food, and water safety in the home [10]. Mother's KAP regarding suboptimal WASH is one of the most critical factors in the transmission of infectious and respiratory tract diseases [11]. Child care without proper hygiene practices makes them far more vulnerable to diseases like diarrhea and cholera; hence, clean water, sanitation, and a safe living environment are vital benefits that every child should enjoy [12].

Recognizing the pivotal position of WASH in child health, the United Nations set forth the ambitious Sustainable Development Goal 6 (SDG 6), *ensuring availability and sustainable management of water and sanitation for all* by 2030 [13]. This includes universal access to safe drinking water, adequate sanitation, and hygiene services, especially for vulnerable and under‐resourced populations. However, at this rate of progress, Bangladesh, specifically its coastal zones, risks failing to hit these targets by the intended deadlines [14, 15].

Household hygiene, particularly WASH, is an important determinant of maternal KAPs. Low maternal knowledge and suboptimal hygiene practices have been associated with higher rates of infection, malnutrition, and child mortality [16]. Although typically considered in isolation, food hygiene is becoming widely accepted as part of WASH, particularly in low‐resource environments where unsafe food practices may further compound health risks for young children [17]. Improving maternal WASH KAP is thus an important avenue to be addressed for improving child health outcomes.

Therefore, on the basis of the KAP model, this study aimed to assess maternal WASH practices in Noakhali, Bangladesh. The study's findings could help understand the existing WASH conditions of households in that particular region, as well as the current level of KAP for WASH among mothers, thereby building evidence to improve the health and well‐being of the people.

## 2 Methods

### 2.1 Study Area

The study was conducted in the “Sonapur” and “Maijdee” areas of Noakhali Sadar Upazila, located in the Noakhali district.

### 2.2 Study Design

The study was a descriptive cross‐sectional study that utilized quantitative data collection methods. It was carried out by targeting households with mothers who had children under the age of five. The sample size was calculated on the basis of the following standard formula for estimates of single proportion:

$$\mathrm{Sample}\;\mathrm{size},n=\frac{Z^{2}p\left(1-p\right)}{d^{2}}$$

where *n* is the required sample size, *Z* is the *Z* statistic for a 95% confidence level (1.96), *p* is the estimated prevalence of the indicator of interest (0.67) [18], and *d* is the margin of error (e.g., 0.05).

$$\mathrm{Using}\;\mathrm{these}\;\mathrm{values}:n=\frac{{\left(1.96\right)}^{2}\times 0.67\times \left(1-0.67\right)}{{\left(0.05\right)}^{2}}=339.8$$

**FIGURE 1:**
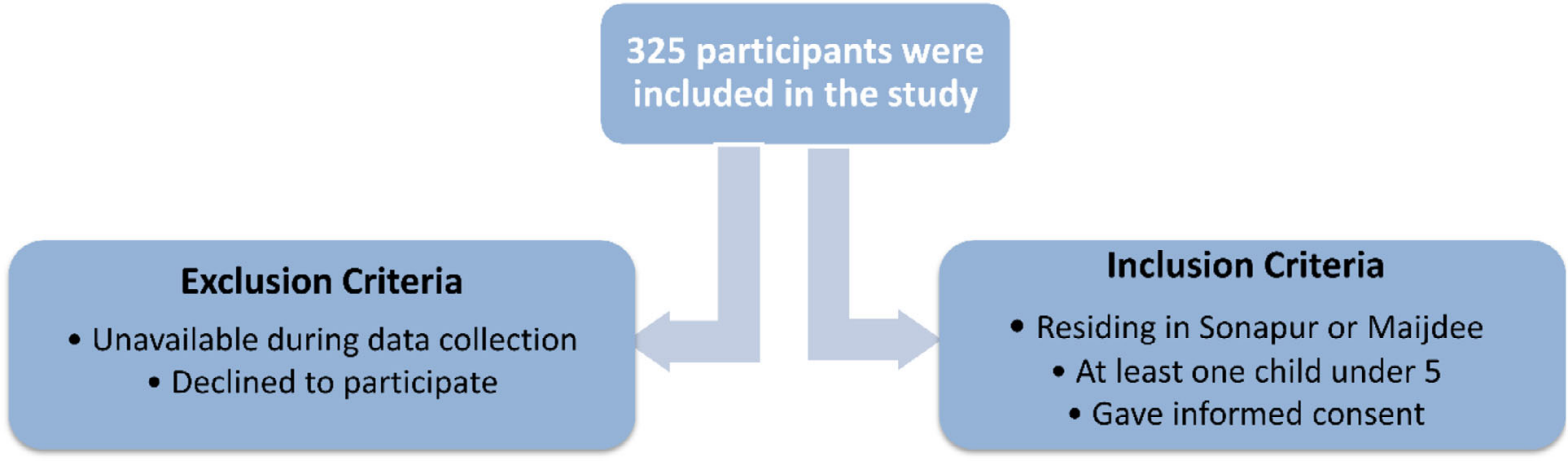
Sampling procedure flowchart.

Thus, the estimated sample size was about 340 mothers. Because of field restrictions, logistics, resource availability, and potential nonresponse, the pragmatic final sample size was determined to be 325 mothers, giving a resultant margin of error of ∼±5.1% at 95% confidence level. A purposive sampling method was employed, and mothers who met the predefined inclusion criteria, which were relevant to the study objectives, were purposively recruited (Figure 1).

### 2.3 Data Collection Methods and Scoring

A pretested, modified, adjusted, and standardized questionnaire was used for data collection through face‐to‐face interviews. The KAP questionnaire was constructed on the basis of a structured KAP framework and guided by the validation process suggested by M. Guad et al. [19], who applied exploratory factor analysis to assess psychometric validity. Public health experts subsequently reviewed it for validity and relevance in context, and a pilot study was performed to assess clarity, reliability, and appropriateness of the tool. Responses were captured using KoBo Toolbox, an Android‐based data collection platform. A total of 43 questions related to knowledge (8 items), attitude (12 items), practice (10 items), and miscellaneous (13 items) were given. Internal consistency was established by Cronbach's alpha (*α* = 0.833), reflecting high reliability. Observation validation was conducted by trained undergraduate students during home visits using a structured checklist. They watched handwashing after defecation and before food preparation, participation in water collection, availability and use of soap with water, cleanliness of water storage containers, and general sanitation behavior inside and outside of the house. Knowledge and practice were scored on a scale of 0 to 1, where “1” was assigned for a positive response and “0” for a negative response. The attitude was scored according to a 5‐point Likert scale, including “0” for strongly disagree, “1” for disagree, “2” for neutral, “3” for *agree*, and “4” for *strongly agree* [20].

#### 2.3.1 Data Analysis

Data were processed and analyzed statistically using SPSS software version 23.0. Descriptive statistics for KAP regarding WASH, along with some socio‐demographic characteristics of the respondents, were performed. A chi‐square test was performed on factors of demographic characteristics of KAP towards WASH. The association between KAP towards WASH, as measured by demographic data, was analyzed using multivariate logistic regression, which determined the odds ratio and 95% confidence interval (CI). A *p* value less than 0.05 was considered statistically significant.

#### 2.3.2 Ethical Statement

The study was approved by the ethics board of Noakhali Science and Technology University. We obtained consent from the participants and thoroughly discussed the study's objectives, as well as its benefits and potential drawbacks.

## 3 Results

Table 1 summarizes the demographic characteristics of mothers who participated in this study. According to the table, it is evident that the majority of mothers were homemakers. It was also found that only 42.8% of mothers had completed secondary and higher education, and half of the families had more than two children. In terms of family income, almost 63% had an annual remuneration of less than 15,000 BDT ($150). After assessing the KAP, it was found that 216 (66.5%) of the mothers had optimal knowledge regarding WASH, whereas the percentage of mothers with an optimal attitude was 81 (24.9%).

**TABLE 1:** Demographic characteristics of respondents.

Characteristics	Frequency	Percentage
**Mother's occupation**		
Housewife	301	92.6
Day labor and others	24	7.4
**Mother's education**		
Illiterate and primary	186	57.2
Secondary and higher	139	42.8
**Family monthly income (BDT)**		
<15,000	204	62.8
≥15,000	121	37.8
**Number of children (in years)**		
≤2	160	49.2
>2	165	50.8

**FIGURE 2:**
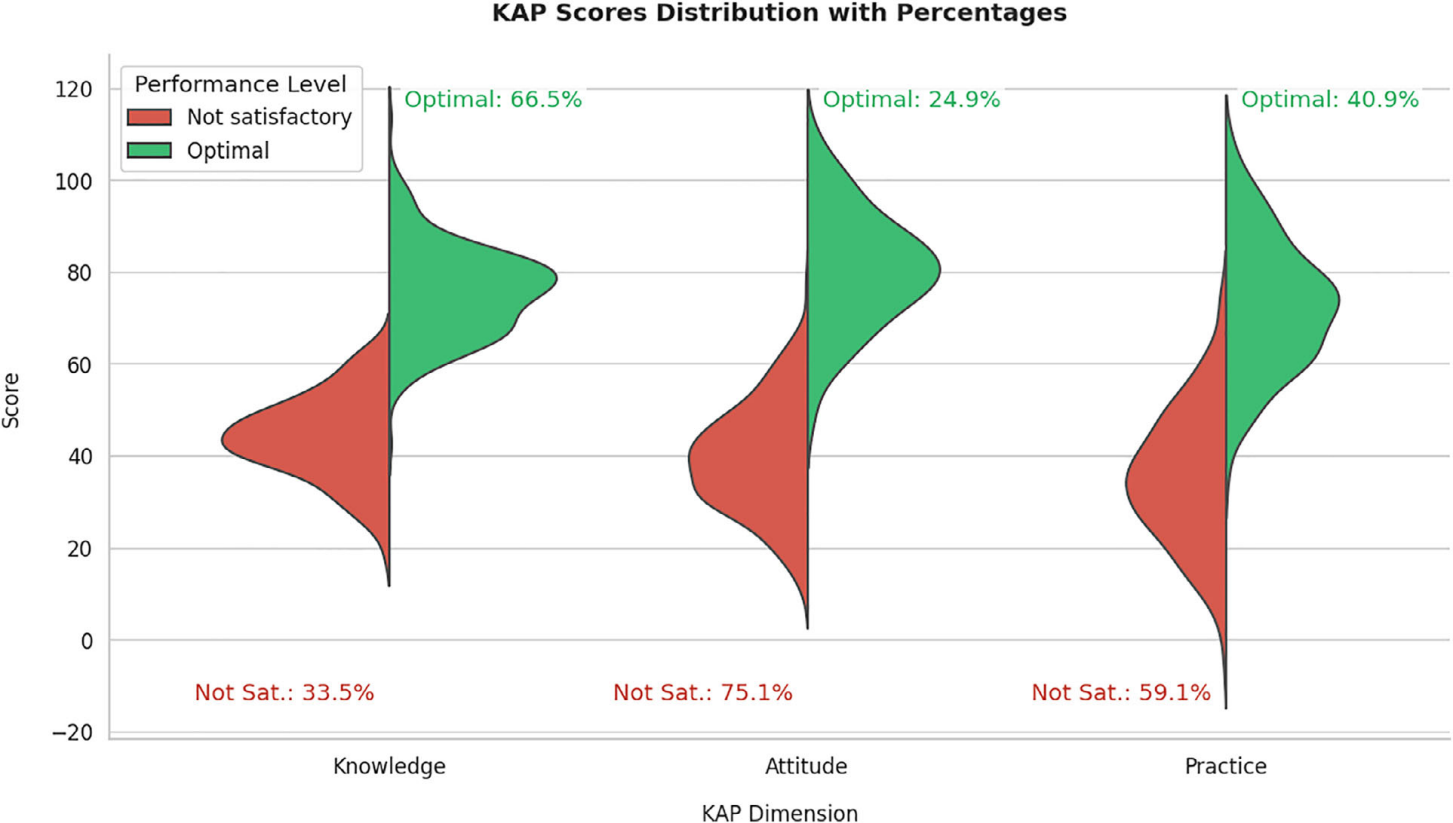
Violin plot of KAP level. KAP, knowledge, attitude, and practice.

The optimal practice regarding WASH among mothers was assessed at the KAP level, as shown in Figure 2, which uses a violin plot to visualize the distribution of participants’ scores for KAPs related to WASH.

### 3.1 Distribution of Responses to Each Question Regarding KAP of WASH

The frequency of WASH‐related KAPs is presented in Tables 2a, 2b, 2c, respectively. Almost half of the participants (41.8%) were unaware of the consequences of liquid waste, and 46.1% were unaware of the prevention mechanism of hand washing. Although most mothers in this study use clean water for hand washing, 33.8% of the participants are unaware of the consequences of not washing their hands. However, the majority of participants correctly recognized that water could be contaminated (90.5%) and unsafe water could cause diarrheal disease (92.9%) (Table 2a).

**TABLE 2a:** Water, sanitation, and hygiene (WASH)‐related knowledge among mothers.

Statement	Response	Frequency (%)
Can unsafe water cause diarrheal diseases?	Yes	92.9
	No	7.1
Can water get contaminated?	Yes	90.5
	No	9.5
Are you using clean water for hand washing?	Yes	83.1
	No	16.9
Do you know about the consequences of liquid waste?	Yes	58.2
	No	41.8
Does animal dung cause disease?	Yes	71.9
	No	20.1
Is a latrine essential and obligatory for every household?	Yes	91.1
	No	8.9
Do you know the prevention mechanism of watery diarrhea?	Yes	53.8
	No	46.1
Do you know the consequences of not washing your hands?	Yes	66.2
	No	33.8

Most participants showed a positive attitude (65.2% agreed, 27.4% strongly agreed) regarding the importance of washing hands after using the latrine, whereas only 26.2% agreed on the importance of washing hands after eating, rather than before eating. Findings revealed that 18.2% disagreed with the assertions that diarrheal disease is transmissible, 3.4% strongly disagreed, and 32% had no opinion (Table 2b).

**TABLE 2b:** Attitude towards water, sanitation, and hygiene (WASH) among mothers.

Statement	Strongly disagree (%)	Disagree (%)	Neutral (%)	Agree (%)	Strongly agree (%)
Clean water consumption is important only when one gets sick	6.8	37.8	23.1	28	4.3
Consumption of safe and enough water can prevent waterborne disease	0.3	6.2	19.7	57.5	16.3
Defecating near water source can cause contamination	0.6	5.2	24.3	57.5	12.3
Boiling water before consumption helps to remove disease‐causing microorganisms	0.9	7.4	21.5	54.8	15.4
The water container must always be cleaned	0.0	6.5	24.0	58.8	10.8
Disposing of liquid waste inside the compound doesn't cause healing problems to seem	4.0	21.8	31.7	36.6	5.8
Diarrheal diseases are caused by poor personal hygiene and sanitation	0.9	6.8	15.1	67.7	9.5
Diarrheal diseases are transmittable	3.4	18.2	32.0	35.7	10.8
Waste can be a breeding site for flies and rodents	0.6	5.5	24.9	62.2	6.8
Washing hands after using the latrine is important	0.3	3.4	3.7	65.2	27.4
Washing hands is more important after eating than before eating	10.8	31.1	28.0	26.2	4.0

Table 2c shows that a large number of households have inappropriate solid waste disposal practices (55.4%), inadequate household waste collection systems (49.8%), improper household waste disposal practices (60.6%), and unprotected sources of water supply (34.8%). Overall, 80.3% of the households have a latrine, and 71.1% of the latrines have hand‐washing facilities. However, only 63.1% use water with soap for hand washing.

**TABLE 2c:** WASH‐related practices among mothers.

Practice	Response	Frequency (%)
Solid waste disposal practice	Inappropriate	55.4
	Appropriate	44.6
Household waste collection	Inappropriate	49.8
	Appropriate	50.2
Household waste disposal	Inappropriate	60.6
	Appropriate	39.4
Have a latrine	Inappropriate	12.6
	Appropriate	87.4
Household latrine with hand washing facilities	No	28.9
	Yes	71.1
Respondent cut nails regularly	No	22.5
	Yes	77.5
Cleanliness of household compound	No	24.0
	Yes	76.0
Latrine utilization	Among those not having a latrine	19.7
	Among those having a latrine	80.3
The material used for hand washing	Water only	36.9
	Water with soap	63.1
Sources of water supply	Unprotected	34.8
	Protected	65.2

### 3.2 Correlation of KAP Regarding WASH With Demographic Characteristics of Mothers

The relationship between demographic characteristics and the mother's KAP is presented in Table 3. Knowledge of the mother was significantly related to number of children and the mother's education and family income. Attitudes and practices regarding WASH were significantly related to family income and the mother's education. According to the table, 57.4% of mothers with more than two children, 46.8% with a family income of ≥15,000 BDT, and 56.5% of mothers who completed secondary and higher education were recorded as having optimal knowledge. In the case of attitude and practice, 69.1% and 65.4% of mothers with secondary and higher education, and 64.2% and 56.4% with income of ≥15,000 BDT, were included with optimal attitude and practice, respectively.

**TABLE 3:** Distribution of respondents according to knowledge, attitude, and practice (KAP) related to water, sanitation, and hygiene (WASH).

Characteristics		Knowledge			Attitude			Practice		
		Not satisfactory	Optimal	*χ* ^2^ *p* value	Not satisfactory	Optimal	*χ* ^2^ *p* value	Not satisfactory	Optimal	*χ* ^2^ *p* value
**Number of children (in years**)	≤2	68 (62.4%)	92 (42.6%)	11.355 0.001*	125 (51.2%)	35 (43.2%)	1.565 0.211	98 (51%)	62 (46.6%)	0.616 0.433
	>2	41 (37.6%)	124 (57.4%)		119 (48.8%)	46 (56.8%)		94 (49%)	71 (53.4%)	
**Family monthly income (BDT)**	<15,000	89 (81.7%)	115 (53.2%)	25.021 0.000*	175 (71.7%)	29 (35.8%)	33.573 0.000*	146 (76%)	58 (43.6%)	35.366 0.000*
	≥15,000	20 (18.3%)	101 (46.8%)		69 (28.3%)	52 (64.2%)		46 (24%)	75 (56.4%)	
**Mother's occupation**	Housewife	99 (90.8%)	202 (93.5%)	0.768 0.381	225 (92.2%)	76 (93.8%)	0.232 0.630	178 (92.7%)	123 (92.5%)	0.006 0.939
	Day labor and others	10 (9.2%)	14 (6.5%)		19 (7.8%)	5 (20.8%)		14 (7.3%)	10 (7.5%)	
**Mother's education**	Illiterate and primary	92 (84.4%)	94 (43.5%)	49.473 0.000*	161 (66.0%)	25 (30.9%)	30.643 0.000*	140 (72.9%)	46 (34.6%)	47.162 0.000*
	Secondary and higher	17 (15.6%)	122 (56.5%)		83 (34.0%)	56 (69.1%)		52 (27.1%)	87 (65.4%)	

^*^ A *p* value of <0.05 is considered statistically significant.

### 3.3 Association of KAP Towards WASH With Mother's Characteristics

The effects of mother's characteristics on WASH‐related KAP are presented in Table 4. Mothers who completed secondary or higher education had 3.3 times higher odds of having good knowledge than those with no or primary education (adjusted odds ratio [AOR] = 3.269, 95% CI: 1.654–6.463, *p* < 0.05). Similarly, mothers with more than two children were more likely to have good knowledge (AOR = 2.534, 95% CI: 1.400–4.570, *p* < 0.05). Household income was not significantly associated with knowledge scores in the adjusted model (AOR = 1.426, *p* > 0.05).

**TABLE 4:** Association of knowledge, attitude, and practice (KAP) related to water, sanitation, and hygiene (WASH) with demographic characteristics.

Characteristics		Knowledge		Attitude		Practice	
		AOR	95% CI	AOR	AOR	95% CI	AOR
**Number of children (in years)**	≤2	Ref		Ref		Ref	
	>2	2.53*	1.40–4.57	1.07	0.60–1.91	0.75	0.44–1.31
**Mother's education**	Illiterate and primary	Ref		Ref		Ref	
	Secondary and higher	3.27*	1.65–6.46	2.47*	1.32–4.60	2.90*	1.66–5.09
**Family Monthly income (BDT)**	<15,000	Ref		Ref		Ref	
	≥15,000	1.43	0.72–2.84	3.09*	1.73–5.51	2.70*	1.54–4.7
**Knowledge**	Not satisfactory level			Ref		Ref	
	Optimal level			7.01*	2.41–20.38	9.62*	4.23–21.88
**Attitude**	Not satisfactory level	Ref				Ref	
	Optimal level	7.59*	2.12–27.20			1.16	0.62–2.20

Abbreviations: AOR, adjusted odds ratio; 95% CI, 95% confidence interval.

^*^ *p* < 0.05 is significant.

Both maternal education and income had a positive effect on attitude scores. Moreover, mothers having higher education levels were 2.5 times more likely to have favorable attitudes (AOR = 2.471, 95% CI: 1.326–4.604, *p* < 0.05), and those from higher income households (≥15,000 BDT) were 3.1 times more likely to have favorable attitude (AOR = 3.090, 95% CI: 1.732–5.511, *p* < 0.05). Children's numbers did not correlate with attitude scores (*p* > 0.05). In addition, knowledge significantly predicted attitude, where mothers with optimal knowledge had seven times higher odds of a positive attitude compared to poor knowledge (AOR = 7.013, 95% CI: 2.41–20.38, *p* < 0.05).

The strongest driver of practice outcomes was knowledge. Mothers with optimal knowledge were approximately 10 times more likely to adopt good hygiene practices (AOR = 9.621, 95% CI: 4.23–21.88, *p* < 0.05), whereas attitude had a statistically nonsignificant impact (*p* > 0.05). Both income and educational attainment continued to be significant predictors of good practice; mothers with higher household income (AOR = 2.70, 95% CI: 1.541–4.733, *p* < 0.05) and secondary education (AOR = 2.901, 95% CI: 1.655–5.087, *p* < 0.05) were more likely to demonstrate appropriate WASH behaviors. Practice scores did not significantly correlate with the number of children (*p* > 0.05).

## 4 Discussion

This study explored maternal KAPs regarding WASH among mothers in rural Noakhali, Bangladesh, revealing essential gaps between knowledge and actual behaviors. Although many mothers demonstrated moderate to good knowledge of WASH principles, translating this knowledge into consistent hygienic practices remains a significant challenge. The findings indicated that 66.5% of the women had optimal knowledge, 24.9% had a positive attitude, and 40.9% demonstrated effective practices regarding WASH. The study revealed that highly educated mothers exhibited significantly greater KAPs related to WASH than illiterate mothers, highlighting the positive impact of academic education on these areas. Additionally, families with lower economic status showed significantly less KAPs concerning WASH.

Regarding sanitation, 87.4% of households surveyed had access to sanitary latrines, which is similar to the findings from Sarail Upazila (80%) [21] and higher than rural homes in India (75%) [22]. But handwashing with soap after defecation was observed among 63.1% in Noakhali—more than in Sarail (33%) [21] and less than soap and water utilized in India (83%) [22]. Open defecation, especially among children, is still a very common practice in developing countries, provoking various health and social risks [23, 24, 25]. The introduction of focused awareness campaigns may replicate the achievements of the Integrated Sanitation Project, launched between 1990 and 1991 in Bangladesh with the assistance of UNICEF [26].

The quality of water sources and the method of storage are also of primary importance. Utilization of protected water sources was reported by only 65.2% of the households, and improper handling or cleaning of storage containers might have contributed to contamination risks. Previous research has demonstrated that uncovered and wide‐mouthed containers are especially susceptible to waterborne pathogens [27, 28]. This practice gap may be due to lack of knowledge, perceived low risk, or economic inability to improve water storage.

Poor waste disposal practices were observed throughout the study area. Most of the households (60.6%) practiced inappropriate method of garbage disposal, and 55.4% had nonfunctional solid waste disposal systems similar to the trend in Northwest of Nigeria [29]. Inappropriate handling and disposal of this waste not only cause harm to the local environment but they also create a risk of the spread of diseases, and dwellers of the community are exposed to an unhealthy environment [30]. Household‐level practices and municipal waste management infrastructure need to be improved to address these concerns.

On a different note, subpar hand‐washing, a common practice in the Noakhali district, may significantly contribute to the spread of germs, pathogens, and viruses, thus resulting in sickness, foodborne illnesses, and nosocomial infections [31]. Hands are one of the significant sources of disease‐causing microorganisms, leading to cross‐contamination with foods, and should be washed appropriately before eating. Despite the significance, only 30.2% of study participants agreed that hand washing before eating is essential, whereas, in another similar type of study, conducted in North India, almost all of the participants (94.85%) reported positive knowledge about the importance of hand washing before and after eating meals [32]. The importance of hand washing after using the latrine is correctly identified by most of the participants (92.6%) in our study as well as in Azraq refugee camp, Jordan (83%); however, an opposite result was found in India in this matter (32%) [22, 33]. On the basis of the findings of our study, 73.8%, 77.2%, and 70.2% of the participants are aware that drinking safe water can prevent waterborne disease, that poor personal hygiene and sanitation are the root causes of diarrheal diseases, and that boiling water before consumption helps to kill disease‐causing microorganisms, respectively. Similar findings (slightly below but matching with our results) were found in another study, conducted in Northern Ethiopia [20]. One of the critical issues in our research was that only 21.6% of the participants knew that diarrheal diseases are transmissible. In contrast, almost all respondents (97.5%) were reported to be aware of this issue in another study conducted in Southern Ethiopia [34]. This finding, or lack of knowledge, suggested the risk of spontaneous spread of diarrheal disease among the larger population in this community.

Additionally, mother's educational level was significantly associated with their WASH‐related KAP in our study. This may be because educated mothers are more aware of the values and concepts related to proper hygiene, sanitation, and safe water, as well as their association with health and disease. Similar findings were reported, as in Sadullapur Upazila [35] and flood‐affected Haor areas [36] of Bangladesh, where maternal education showed significant impact on their knowledge regarding sanitation, safe water, and water hygiene practices.

Family income was identified as a predictor of WASH‐related KAPs in the current study. This result corresponds with a considerable amount of research which demonstrates a positive association between increased socioeconomic status and better hygiene practices in other settings. For instance, reports from Bangladesh [37], India [38], and Nicaragua [39] find that richer families are more inclined to wash their hands frequently, live in relatively cleaner settings, and enjoy access to sanitation and hygiene goods and services, such as soap and safe water, than poorer households. Financial stability allows families to invest in vital WASH infrastructure such as private latrines, kitchens, or water collection points, and this in turn supports better hygiene behavior.

Evidence from several countries in South Asia supports the trends visible in this study, demonstrating that maternal education, income, and social norms have an effect on WASH practices. In all these studies, although awareness on hand hygiene was high, especially after defecation, consistent practices prior to cooking, eating, and feeding children were among the lowest [40, 41, 42, 43, 44]. This knowledge‐behavior gap was explained as influenced by obstacles like the habit of using water only, social norms, the availability of soap, and the burden on caregivers [38, 40, 42, 45]. In line with our results, inadequate waste disposal and unsafe water storage behaviors were reported also in cases where access to sanitation facilities was available, revealing a continuity of environmental hazards [40, 46]. Effect of maternal socio‐demographic factors on WASH Information on WASH education and WASH practices forms the basis of the health‐promoting practices on a family and community level, which is influenced by maternal education, together with income level. Almost all studies indicated the association of mothers’ education and higher income with WASH being on the positive side, thus confirming also our result that these two variables significantly predicted better KAPs [38, 44, 45]. Such regional similarities warrant a strategy of interventions that combines health education, behavior change, and infrastructure implementation that are sensitive to local socio‐cultural conditions.

Taken together, these findings suggest that knowledge alone is insufficient to guarantee safe hygiene practices. This illustrates the challenge of behavior change, where people may know what to do but struggle to put that knowledge into practice for reasons like motivation, environment, or strong habits. Effective public health strategies should combine targeted education and behavior change communication with accessible infrastructure improvements. Collaborative efforts among government agencies, community organizations, and local leaders are essential to bridge the gap between awareness and practice and to reduce the burden of preventable diseases in rural Bangladeshi communities.

### 4.1 Strengths and Limitations

This study offers valuable, context‐specific insights into maternal KAPs related to WASH among women in a coastal region of Bangladesh. A key strength lies in the use of a structured KAP framework, benchmarked against national and international standards, which enhances both the internal validity and external relevance of the findings. Furthermore, the study's comprehensive approach, encompassing multiple WASH components including water quality, sanitation facilities, hand hygiene, and waste disposal, provides a holistic assessment of behavioral and environmental factors influencing maternal and child health.

Nevertheless, several limitations should be acknowledged. The cross‐sectional design restricts the ability to infer causal relationships between demographic factors and WASH practices. A second study limitation is the purposive sampling strategy given the fieldwork constraints. Although it guarantees that there are related individuals, it can also introduce bias, which restricts the generalization of the results. Additionally, reliance on self‐reported data introduces potential for recall bias and social desirability bias, which could affect the accuracy of reported behaviors. Finally, the geographic focus on the Noakhali district may limit the generalizability of the findings to other regions of Bangladesh, particularly urban or non‐coastal settings.

## 5 Conclusion

This study highlights significant gaps in maternal KAPs related to WASH among rural mothers in Noakhali, Bangladesh. Higher maternal education and household income emerged as essential determinants of better hygiene practices, underscoring the critical role of social determinants in shaping public health outcomes.

Key behavioral shortcomings, including inadequate handwashing at mealtimes, limited knowledge of proper waste disposal, and only moderate understanding of diarrheal disease transmission, suggest that knowledge alone is insufficient to translate into consistent hygienic practices. These findings emphasize the need for holistic, community‐based interventions that combine infrastructure development with behavior change communication strategies.

Addressing the burden of poor WASH outcomes in similar rural settings will require sustained investment in women's education, targeted health promotion initiatives, and collaborative efforts between government agencies and community stakeholders. Advancing health in these areas has the potential to significantly reduce the prevalence of preventable diseases and improve overall community well‐being.

## Author Contributions

**Prantu Sen:** Conceptualization, Methodology, Formal analysis, Writing ‐ review & editing, Writing ‐ original draft, Software; **Mohammad Asadul Habib:** Conceptualization, Methodology, Software, Data curation, Investigation, Formal analysis, Supervision, Writing ‐ original draft, Writing ‐ review & editing; **Tanjina Rahman:** Supervision, Writing ‐ review & editing, Investigation, Methodology, Visualization; **Akibul Islam Chowdhury:** Formal analysis, Validation, Software, Methodology, Writing ‐ review & editing, Data curation; **Kazi Muhammad Rezaul Karim:** Writing ‐ review & editing, Supervision, Methodology, Software, Validation

## Conflicts of Interest

The authors declare no conflicts of interest.

## Data Availability

Data will be made available on request.
